# Draft Genome Sequence of Proteus mirabilis Strain UMB0038, Isolated from the Female Bladder

**DOI:** 10.1128/MRA.00401-20

**Published:** 2020-05-21

**Authors:** Noreen Gallian, Taylor Miller-Ensminger, Adelina Voukadinova, Alan J. Wolfe, Catherine Putonti

**Affiliations:** a Department of Biology, Loyola University Chicago, Chicago, Illinois, USA; b Bioinformatics Program, Loyola University Chicago, Chicago, Illinois, USA; c Department of Microbiology and Immunology, Stritch School of Medicine, Loyola University Chicago, Maywood, Illinois, USA; d Department of Computer Science, Loyola University Chicago, Chicago, Illinois, USA; University of Maryland School of Medicine

## Abstract

Proteus mirabilis is a Gram-negative motile and rod-shaped bacterium that is a common pathogen of the urinary tract. Here, we report the draft genome sequence of P. mirabilis UMB0038, which was isolated from a woman without lower urinary tract symptoms.

## ANNOUNCEMENT

Proteus mirabilis is well known for its prominent flagella, allowing it to move across surfaces. Unique virulence factors such as urease production, fimbriae, iron and zinc acquisition, toxins, and biofilm formation allow it to overtake many other species with which it comes into contact, allowing it to be a common pathogen within the urinary tract (1). P. mirabilis is commonly seen in complicated urinary tract infection (UTI) cases that include long-term catheterization (2). Catheter-associated UTIs are frequently caused by P. mirabilis biofilm formation on the catheter surface, blocking urine flow. Therefore, much research has gone into methods for inhibiting biofilm formation (see reviews [3, 4]). Here, we present the draft genome sequence of a P. mirabilis strain, UMB0038, isolated from a catheterized urine sample obtained from a “healthy” woman without lower urinary tract symptoms.

P. mirabilis was collected as part of prior institutional review board (IRB)-approved studies (5–8) using the expanded quantitative urine culture (EQUC) protocol (8). The genus and species for this isolate were determined through matrix-assisted laser desorption ionization–time of flight (MALDI-TOF) mass spectrometry (8) prior to storage at −80°C. From the freezer stock, the P. mirabilis isolate was streaked onto a Columbia nalidixic acid (CNA) plate and incubated for 24 h at 35°C in 5% CO_2_. A single colony was selected from the plate, added to liquid tryptic soy broth (TSB) and incubated for 48 h at 37°C with shaking. DNA was extracted using the Qiagen DNeasy blood and tissue kit following the protocol for Gram-positive bacteria with minor modification. An adjusted amount of 230 μl of lysis buffer (180 μl of 20 mM Tris-Cl, 2 mM sodium EDTA, and 1.2% Triton X-100 and 50 μl of lysozyme) was used, with an incubation time of 10 min after the addition of buffer AL. DNA was quantified using a Qubit fluorometer and then sent to the Microbial Genome Sequencing Center (MiGS) at the University of Pittsburgh for sequencing on the Illumina NextSeq 550 platform. There the DNA was enzymatically fragmented using an Illumina tagmentation enzyme, and indices were attached using PCR. A total of 1,565,124 pairs of 150-bp reads were produced. Raw reads were trimmed using Sickle v1.33 (https://github.com/najoshi/sickle) and assembled using SPAdes v3.13.0 with the only-assembler option for k values of 55, 77, 99, and 127 (9). The genome coverage for the assembly was 103× and was calculated using BBMap v38.47 (https://sourceforge.net/projects/bbmap). Genome quality assessment and annotation were conducted with PATRIC v3.6.3 (10). The NCBI Prokaryotic Genome Annotation Pipeline (PGAP) v4.8 (11) also was used to annotate the genome sequence. Default parameters were used for each software tool unless previously stated.

The P. mirabilis UMB0038 draft genome was assembled into 57 contigs. It is 3,946,388 bp long with an *N*_50_ value of 164,741 bp and a GC content of 38.6%. The size and GC content are similar to those of other strains of this species in GenBank. PGAP identified 3,533 protein-coding genes in the genome assembly. PHASTER (12) found three incomplete phages and one intact phage within this genome. The intact phage is of particular interest, because very few P. mirabilis-infecting phages are known (13) and phages are one strategy currently being explored to combat P. mirabilis biofilms (3). Additional analysis of this strain and genome will provide insight into how unique virulence factors contribute to UTIs.

### Data availability.

This whole-genome shotgun project has been deposited in GenBank under the accession no. JAAUWO000000000. The version described in this paper is the first version, JAAUWO010000000. The raw sequencing reads have been deposited in the SRA under the accession no. SRR11441037.
